# Progression-free survival outcomes of PARP inhibitors in ovarian cancer: an exploratory analysis of treatment heterogeneity based on organ vulnerability

**DOI:** 10.3389/fonc.2026.1768842

**Published:** 2026-07-03

**Authors:** Xing Zhou, Xi’an Xiong, Zhen Yang, Zhongping Cao, Qianxi Ni

**Affiliations:** 1 The Affiliated Cancer Hospital of Xiangya School of Medicine, Central South University/Hunan Cancer Hospital, Changsha, China; 2 Oncology Department of Xiangya Hospital, Central South University, Changsha, China; 3 Department of Oncology of the Second Xiangya Hospital, Central South University, Changsha, China

**Keywords:** causal inference, heterogeneous treatment effects, organ vulnerability, ovarian cancers, PARP inhibitors, real-world evidence

## Abstract

**Background:**

PARP inhibitors are standard maintenance therapies for ovarian cancer (OC), but evidence regarding how baseline physiological vulnerability relates to heterogeneity in comparative treatment effectiveness—beyond molecular biomarkers—remains limited. This study applied a causal inference framework, benchmarked against established molecular associations, to explore whether organ vulnerability is associated with variation in progression-free survival (PFS) between PARP inhibitors.

**Methods:**

In this retrospective study of 604 OC patients, we constructed an interpretable 0–3 organ vulnerability score (OVS) using routine pre-treatment laboratory indicators reflecting physiological reserve. A benchmark analysis in 280 patients with complete BRCA status evaluated whether the framework could reproduce established prognostic associations under real-world conditions. G-computation–based counterfactual survival standardization and causal forest analyses were applied to the full cohort to explore OVS-associated treatment heterogeneity, with directional reproducibility assessed in an independent external cohort (n = 58).

**Results:**

The estimated association between BRCA mutation status and PFS was directionally consistent with prior clinical evidence (HR = 0.685, 95% CI: 0.490–0.959; P = 0.028). In the full cohort, a significant treatment-by-OVS interaction was observed (interaction HR = 0.488, 95% CI: 0.293–0.813; P = 0.006). Among patients with low vulnerability (OVS 0–1, n = 479), olaparib was associated with a modest PFS advantage over niraparib (HR = 0.744, P = 0.030). In patients with high vulnerability (OVS 2–3, n = 125), the estimated relative benefit associated with olaparib was more pronounced (HR = 0.363, P < 0.001); however, these estimates are model-dependent and should not be interpreted as definitive comparisons. The directional heterogeneity pattern was qualitatively reproduced in the external cohort. Exploratory heterogeneity analysis further identified individual-level variation in estimated treatment response, including a subset with estimated relative benefit favoring niraparib.

**Conclusions:**

This retrospective exploratory analysis suggests that baseline physiological vulnerability may be associated with heterogeneity in the relative effectiveness of PARP inhibitors. These findings are hypothesis-generating and lay the groundwork for prospective investigation into physiological vulnerability as a correlate of heterogeneous treatment response in real-world oncology populations.

## 1 Introduction

Ovarian cancer (OC), one of the most lethal gynecological malignancies, is characterized by insidious onset and rapid progression. Approximately 70% of patients are diagnosed at an advanced stage, with a five-year survival rate persistently below 40% (1, 2). The advent of poly (ADP-ribose) polymerase (PARP) inhibitors has significantly improved progression-free survival (PFS) in OC. These agents have become standard maintenance therapy for patients with BRCA mutations or homologous recombination deficiency (HRD) positivity, as well as for platinum-sensitive recurrence or first-line maintenance settings (3–5). Currently, olaparib and niraparib are widely used globally, with efficacy thoroughly validated in randomized controlled trials (RCTs) (1, 2, 4, 5). However, in real-world practice, substantial heterogeneity in treatment benefit is observed—some patients, despite harboring favorable molecular markers, do not achieve the expected benefit due to early discontinuation, dose modification, or rapid disease progression (6–8).

Previous studies have primarily focused on molecular-level predictors. BRCA mutation status and HRD score have been established as key biomarkers for PARP inhibitor efficacy and are included in multiple international guidelines (9–12). Notably, approved indications differ across agents: olaparib’s initial indication primarily targeted BRCA-mutated patients based on trials such as SOLO-1 (1), whereas niraparib demonstrated PFS benefit in the broader platinum-sensitive population—including HRD-negative patients—in the PRIMA trial (2). However, this molecular marker-centric approach has important limitations. Molecular testing coverage remains limited in resource-constrained settings, leaving a substantial proportion of patients without biomarker information. Moreover, molecular status reflects tumor sensitivity but does not capture physiological tolerance to treatment. Different PARP inhibitors exhibit distinct toxicity profiles: niraparib was associated with a higher incidence of grade 3/4 thrombocytopenia (approximately 34%) in the NOVA trial (5), whereas olaparib’s hematologic toxicity is comparatively milder (4). This disparity suggests that in patients with compromised organ function reserves, differences in toxicity profiles may influence relative treatment benefit. Yet systematic evaluations of how baseline physiological status modifies the relative efficacy of different PARP inhibitors remain limited.

Despite increasing availability of real-world data, most analyses remain focused on prognostic modeling—predicting individual PFS risk using machine learning or related approaches (13)—rather than addressing the comparative question central to clinical decision-making: for a given patient, what is the relative PFS benefit under different treatment strategies? Addressing this question requires a causal inference framework capable of estimating treatment effects under alternative strategies and identifying effect modifiers (14–16).

We therefore constructed an interpretable Organ Vulnerability Score (OVS) using routine laboratory markers as proxies of baseline physiological reserve (17–20), and applied G-computation–based counterfactual survival standardization and causal forest approaches to explore heterogeneous treatment effects (HTE). The objective was not prognostic prediction but rather to examine whether baseline physiological reserve may modify the relative effectiveness of different PARP inhibitors. To evaluate the analytical framework’s capacity to recover established treatment-response patterns, we first conducted a benchmark analysis in 280 patients with known BRCA status, using the well-established association between BRCA mutation and PARP inhibitor response (1, 2, 4, 5) as a plausibility reference. We then extended the analysis to the full cohort of 604 patients to examine the interaction between OVS and treatment type, with robustness evaluated through prespecified sensitivity analyses (8). Overall, this study presents an exploratory causal inference framework for examining whether baseline physiological reserve may contribute to heterogeneity in PARP inhibitor treatment effects, particularly in settings with incomplete molecular characterization.

## 2 Materials and methods

This study employed a target trial emulation framework to evaluate causal effects of different PARP inhibitors on PFS in a real-world observational setting, while controlling for confounding and exploring the modifying role of baseline physiological status on heterogeneous treatment effects. By explicitly defining study protocol elements—including study population, treatment variables, outcome measures, and strategies for handling concomitant events—this framework provides a structured foundation for causal inference in observational studies (14). To ensure clinical relevance and methodological transparency, the study followed a modified intention-to-treat (ITT) principle, incorporating all patients who initiated PARP inhibitor therapy and assigning them based on the actual drug received (21).

### 2.1 Study population and eligibility criteria

The study cohort comprised OC patients treated with PARP inhibitors between August 2018 and November 2023 (n = 604). Inclusion criteria were: (i) histologically confirmed OC; (ii) partial or complete remission after at least one line of platinum-based chemotherapy; (iii) PARP inhibitor maintenance therapy with olaparib or niraparib; and (iv) availability of baseline covariate information required for causal adjustment. Data were sourced from the medical databases of Hunan Cancer Hospital, China, using a standardized data collection protocol, and encompassed demographic characteristics, tumor biology, treatment history, and comprehensive pre-treatment laboratory parameters. Although BRCA mutation and HRD status are important biomarkers, they were not used as exclusion criteria due to limited clinical testing coverage; all patients were included regardless of known BRCA/HRD status to reflect real-world practice. Patient enrollment was defined as the date of PARP inhibitor initiation, with all baseline covariates measured prior to this date. The study protocol was approved by the hospital’s ethics committee (Ethical Code Number: KYJJ-2023-302), and written informed consent was obtained from all participants. All analyses were performed on de-identified data (22, 23).

**Figure 1:**
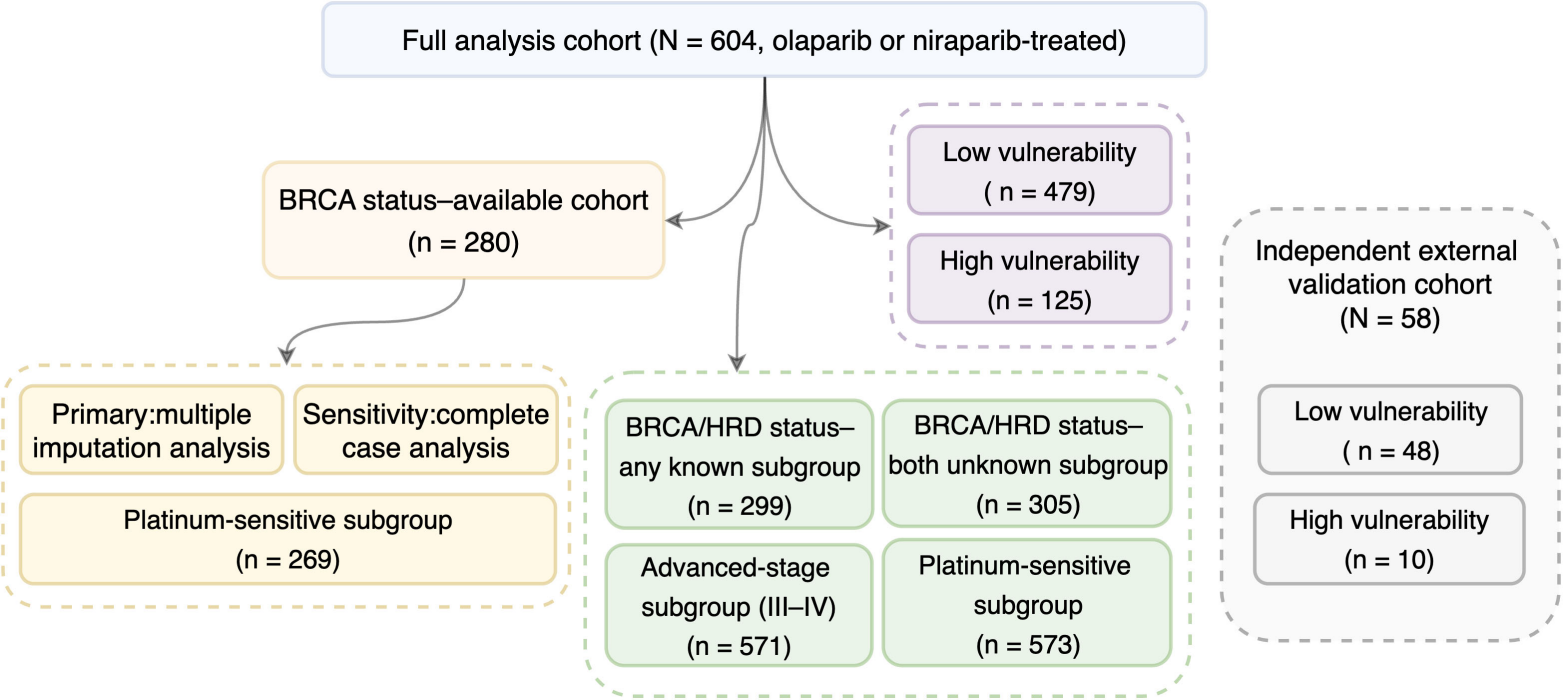
Study flowchart and subgroup analysis strategy.

**Figure 1** illustrates the derivation of the analyzable cohort (n = 604). Two primary analytical branches were defined: the benchmark subcohort with documented BRCA status (n = 280), used to assess the causal association between BRCA mutation and PFS; and the full cohort stratified by OVS, used to evaluate whether baseline organ vulnerability modifies the relative efficacy of different PARP inhibitors. Sensitivity analyses were conducted across multiple predefined subgroups. The independent external validation cohort (n = 58) was collected from two tertiary academic centers and analyzed using the same prespecified OVS framework to assess directional reproducibility of the primary findings.

### 2.2 Target trial protocol specification

#### 2.2.1 Treatment strategies

Treatment strategy was defined according to the PARP inhibitor used for maintenance therapy (olaparib or niraparib). Because niraparib is approved for a broader platinum-sensitive population in China, whereas olaparib was initially indicated primarily for BRCA-mutated patients (2), treatment allocation in routine practice may reflect underlying differences in patient characteristics and prescribing patterns. Accordingly, treatment assignment was treated as a potential source of confounding and adjusted for in the causal analyses. In regression-based models, niraparib was treated as the reference category, such that hazard ratios below 1.0 indicate relative benefit associated with olaparib.

#### 2.2.2 Outcomes and follow-up

PFS was defined as the time from PARP inhibitor initiation to the first occurrence of radiographically confirmed disease progression (RECIST v1.1) or death from any cause. Patients without an event were censored at the data collection cutoff date or at loss to follow-up.

#### 2.2.3 Intercurrent events

Given the absence of systematically recorded toxicity events and dose adjustment logs in real-world electronic health records, toxicity events were not directly modeled. Instead, baseline organ function status was used as a proxy for patients’ intrinsic susceptibility to treatment-related toxicity, indirectly reflecting physiological capacity to maintain full-dose therapy. A treatment-policy strategy analogous to modified ITT was adopted: patients were analyzed according to the initial PARP inhibitor assigned at time zero, regardless of subsequent dose reductions or interruptions. This approach ensures that the estimand reflects the real-world association under a treatment-policy strategy.

#### 2.2.4 Baseline covariates (W)

Baseline clinical and laboratory information was collected prior to PARP inhibitor initiation, including demographic characteristics, disease status, treatment history, molecular testing results were available, and routine pre-treatment laboratory measurements. Covariate selection for causal adjustment was guided by a prespecified DAG-informed framework. The adjustment set was restricted to a minimal sufficient set of pre-treatment confounders jointly associated with treatment allocation and PFS, including age, disease stage, treatment line, and molecular status (BRCA/HRD where available). Variables considered potential mediators, downstream consequences, or components of the OVS construct were intentionally excluded to preserve interpretability of the heterogeneous treatment effect estimand and minimize collider bias.

#### 2.2.5 Effect modifiers

OVS was prespecified as the primary effect modifier and designed to represent baseline physiological reserve independently of tumor-related characteristics. The construct was based on three clinically relevant organ systems involved in treatment tolerance and drug metabolism: hematologic, hepatic, and renal function (24–29). Thresholds were defined using established clinical reference standards, including CTCAE v5.0 (30), WS/T 404–2012 reference intervals (31), and conventional hepatic reserve assessment principles derived from the Child–Pugh framework (32). Hematologic vulnerability was defined by hemoglobin <110 g/L, platelets <150×10⁹/L, or absolute neutrophil count <1.5×10⁹/L. Hepatic vulnerability was defined by albumin <35 g/L, total bilirubin >17.1 μmol/L, or AST >40 U/L. Renal vulnerability was defined by creatinine >90 μmol/L or uric acid >360 μmol/L in females (33). Each organ system contributed 1 point if any corresponding indicator exceeded the predefined threshold, yielding a total OVS ranging from 0 to 3. An equal-weighting strategy was adopted based on parsimony and the conceptual assumption that impairment in any major physiological system may represent a clinically relevant limitation in treatment tolerance (34, 35).

OVS was designed as a clinically interpretable composite physiological stratification variable rather than a prognostic prediction model. Accordingly, it was modeled exclusively as an effect modifier to evaluate heterogeneity in the relative treatment effects of olaparib versus niraparib (36–38). Components of the OVS construct were intentionally excluded from the adjustment set to avoid overadjustment and preserve interpretability of the heterogeneous treatment effect estimand.

### 2.3 Identification assumptions and causal estimands

This study addressed two objectives within a causal inference framework: (i) to assess whether the analytical pipeline could reproduce the established association between BRCA mutation status and PFS in a biomarker-defined subcohort; and (ii) to evaluate whether baseline organ vulnerability modified the relative effectiveness of different PARP inhibitors in the full cohort, including patients with incomplete molecular profiling. Treatment assignment was not randomized and may have been influenced by multiple clinical and non-clinical factors; prescribing patterns were therefore treated as a potential source of confounding and addressed through prespecified confounder adjustment strategies.

#### 2.3.1 Identification assumptions

To formalize the assumed data-generating process, a directed acyclic graph (DAG) was used to identify a minimally sufficient adjustment set of baseline covariates (W) (39–43). Standard identification assumptions for observational causal inference—conditional exchangeability, positivity, and consistency—were invoked (16, 17).

Conditional exchangeability assumes that, given the observed baseline covariates (W), treatment assignment is independent of potential outcomes. W included age, disease stage, treatment history, and molecular markers where available. Residual confounding from unmeasured factors—such as physician prescribing preferences or healthcare access—cannot be excluded; E-value analysis was performed to assess sensitivity to unmeasured confounding.

The positivity assumption requires that patients with similar baseline characteristics have a non-zero probability of receiving either treatment, and was empirically evaluated through propensity score distributional overlap between treatment groups.

The consistency assumption implies that the observed outcome under the received treatment corresponds to the potential outcome under that treatment strategy, and was considered plausible given the clearly defined treatment strategies.

**Figure 2:**
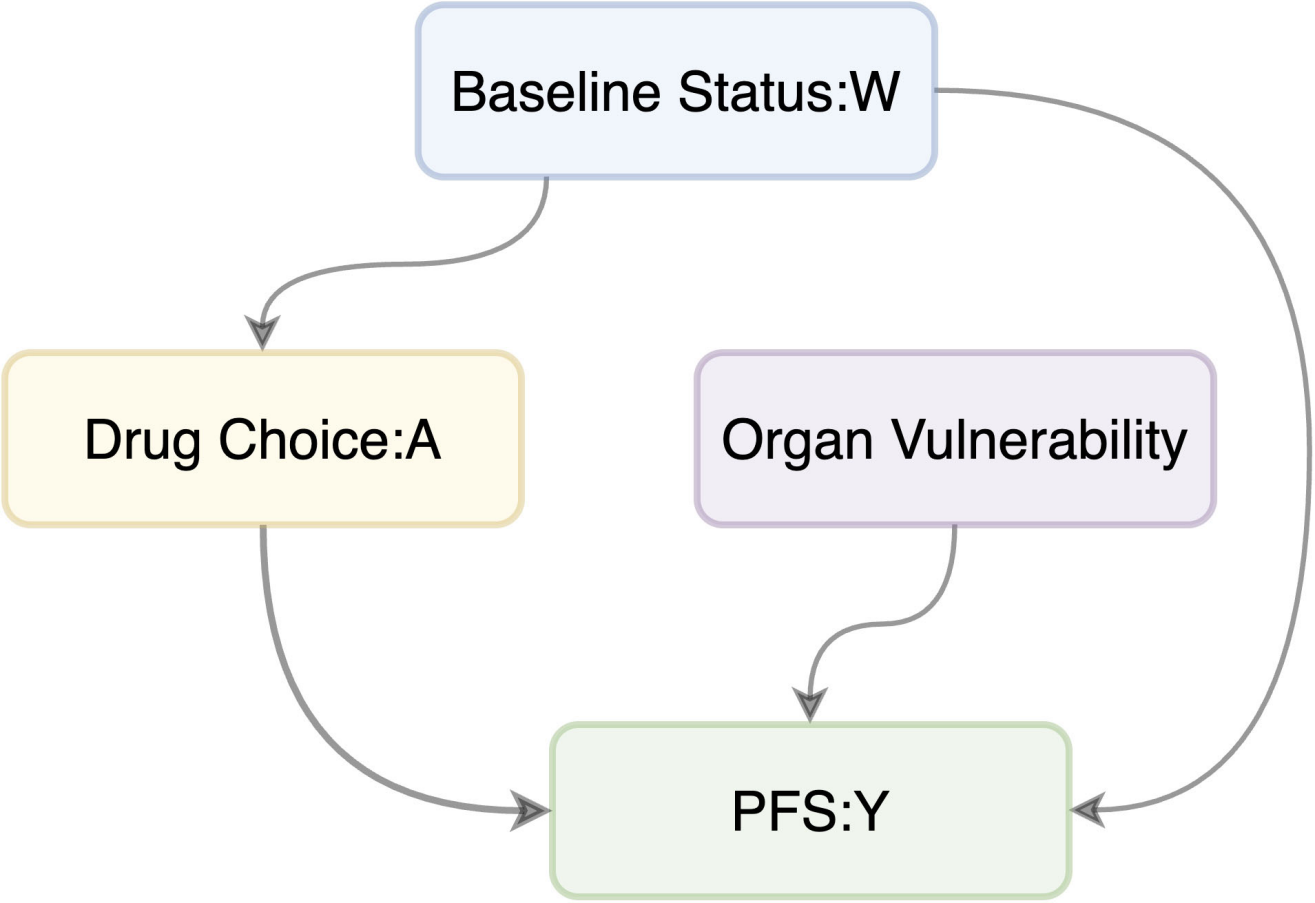
DAG illustrating the hypothesized causal framework of the study. Baseline covariates (W; e.g., age, stage, BRCA/HRD status, and treatment history) may influence both PARP inhibitor selection (*A*) and PFS (*Y*). OVS was conceptualized as a pre-treatment physiological stratification variable and evaluated as a prespecified effect modifier of the treatment–PFS association.

**Figure 2** summarizes the hypothesized causal framework and prespecified relationships among baseline covariates, treatment assignment, OVS, and PFS.

Core adjustment variables—age, FIGO stage, treatment line, and molecular status—were retained in the primary adjustment set based on established relevance to both treatment allocation and prognosis (39, 40). Variables with extremely low prevalence were excluded from the primary model due to limited statistical support but were evaluated in sensitivity analyses. All estimated associations should be interpreted within the context of these assumptions and the inherent limitations of observational data.

#### 2.3.2 Benchmark validation: BRCA status–PFS relationship

To evaluate the internal validity of the proposed causal inference pipeline, a benchmark analysis was conducted within a subcohort of 280 patients with available BRCA mutation data. BRCA mutation status was specified as a binary exposure variable (*A*) and PFS as the outcome (*Y*). The objective was not to imply a manipulable intervention on BRCA status, but to assess whether the analytical framework could recover well-established survival patterns under controlled confounding, serving as an empirical calibration step for subsequent analyses (16). The benchmark framework used a simplified causal structure in which BRCA status was treated as a baseline biological exposure without treatment allocation mechanisms or exposure–modifier interactions; identification relied on adjustment for measured baseline covariates satisfying the backdoor criterion.

- Confounder Selection and Propensity Score Estimation: Baseline covariates (**W**) were selected based on the simplified DAG, including age, FIGO stage, and number of prior platinum-based chemotherapy lines. A logistic regression model (scikit-learn LogisticRegression, solver = “liblinear”, max_iter = 1000) was used to estimate the propensity score P(A=1|W), with categorical variables encoded as dummy variables prior to model fitting. Stabilized inverse probability of treatment weights (IPTW) were computed as $S_{w}=\frac{P(A)}{P(A|W)}$, where P(A) denotes the marginal probability of BRCA mutation. Weights were truncated at the 1st and 99th percentiles to improve numerical stability and mitigate the influence of extreme weights (44–46). Positivity was assessed through propensity score distributional overlap between exposure groups.
- Robustness and Sensitivity Analyses: Multiple imputation (MI) was prespecified to address limited missingness in baseline covariates. Benchmark validation was restricted to the subcohort with fully observed BRCA mutation status (n = 280); no imputation of BRCA status was performed. Missingness was limited to a small number of covariates (primarily age, FIGO stage, and treatment line) and was addressed using iterative multivariate imputation (scikit-learn IterativeImputer, max_iter = 50, sample_posterior = True), with random seeds incrementally assigned across five imputed datasets (42 through 46) to ensure reproducibility. Survival outcomes were excluded from the imputation model to avoid outcome-informed imputation. Five imputed datasets were analyzed independently using the full causal pipeline and pooled using Rubin’s rules (47). Three complementary analytical strategies were implemented: (i) primary analysis using MI-based pooled estimates; (ii) CCA restricted to patients with fully observed covariates; and (iii) sensitivity analysis in platinum-sensitive patients. Concordance across strategies was interpreted as supportive evidence for robustness.
- Weighted Survival Association Estimation: For each imputed dataset, IPTW-weighted Cox proportional hazards models (lifelines CoxPHFitter) were fitted with PFS as the outcome, including only the exposure variable (BRCA status) and stabilized weights, consistent with a marginal structural modeling framework. Robust sandwich variance estimators were used to account for weighting-induced uncertainty. P-values for CCA estimates were derived from the Wald test; P-values for MI-based estimates were derived from Rubin’s rules pooling. For covariate balance assessment, ordered categorical variables (FIGO stage and treatment line) were expanded into indicator variables to enable category-level SMD evaluation, consistent with standard practice for weighted observational analyses; in outcome models, these variables were retained as ordered integer-coded covariates to preserve model stability (48, 49). Final pooled HRs and 95% CIs were derived using Rubin’s rules.

#### 2.3.3 Evaluation of treatment effect heterogeneity via OVS

The primary causal estimand was defined as the Conditional Average Treatment Effect (CATE) within the potential outcome framework (39). Let Y(1) and Y(0) denote the potential PFS outcomes if a patient were assigned to niraparib (A=1) or olaparib (A=0), respectively; negative CATE values and hazard ratios below 1.0 throughout indicate relatively more favorable estimated outcomes under olaparib compared with niraparib. The OVS-modified CATE is defined as: τ(v)=E[Y(1)−Y(0)∣V=v,W], where *V* represents the OVS and W denotes the baseline confounders. This estimand represents a model-based contrast in survival outcomes between the two treatments across levels of physiological status. To estimate τ(v) and characterize its variation across OVS levels, we implemented a dual-modeling approach:

(I) Model-based Counterfactual Standardization: A model-based counterfactual standardization approach, conceptually analogous to G-computation, was implemented to estimate adjusted survival probabilities across OVS strata. The procedure followed three steps:

(i) Nuisance Model Fitting: Stabilized IPTW were computed using the same logistic regression–based propensity score estimation approach described in Section 2.3.2, incorporating the full baseline covariate set (W), with weights truncated at the 1st and 99th percentiles. A weighted Cox proportional hazards model (lifelines CoxPHFitter) was then estimated on the observed data. The model included the treatment indicator (*A*), OVS, their interaction term (*A* × OVS), and the baseline covariate set (W), with robust sandwich variance estimation to account for weighting uncertainty.

(ii) Counterfactual Projection: For every individual in the observed dataset, the fitted Cox model was used to generate two counterfactual survival functions— S(t|A=1, W , V) and S(t|A=0, W , V)—by setting the treatment indicator to *A* = 1 (niraparib) and *A* = 0 (olaparib) in turn while holding all other covariates at their observed values. This individual-level counterfactual prediction step constitutes the core of the G-computation–analogous standardization procedure.

(iii) Marginal Standardization: Individual-level counterfactual survival curves were averaged within each OVS stratum to generate standardized marginal survival estimates for each treatment arm, allowing descriptive comparison of standardized treatment-specific survival trajectories across vulnerability subgroups under the identifying assumptions of the causal framework.

(II) Machine Learning–Based Exploratory Heterogeneity Detection:

To explore potential non-linear variation in estimated treatment response without pre-specifying subgroup structure, we applied the Causal Forest algorithm (econml.grf.CausalForest) as an exploratory machine learning–based heterogeneity detection tool. Because the outcome was specified as observed PFS duration (in months) rather than a censoring-adjusted survival estimand, this analysis should be interpreted as an exploratory assessment of differential treatment response patterns rather than a confirmatory causal estimation of survival treatment effects.

(i) Model Configuration: Model Configuration: To partially account for informative censoring, inverse probability of censoring weights (IPCW) was estimated using treatment-stratified Kaplan–Meier estimators of the censoring time distribution and applied as sample weights in the causal forest. The forest was trained with 300 trees, a minimum leaf size of 15, a maximum depth of 10, and subsampling for honesty. OVS was included as a candidate continuous splitting variable alongside the molecular and clinical covariate set (W). Additional hyperparameters included: sample fraction = 0.5 (default); number of features considered at each split = square root of total features (default). All random seeds were fixed at 42 for reproducibility.

(ii) Handling of Missing Molecular Data: Missing BRCA and HRD data were handled using a predefined “Unknown” category strategy, encoding missing values as a separate category rather than imputing them, reflecting the potentially informative nature of molecular testing missingness in real-world settings (50–52). Accordingly, BRCA/HRD variables were excluded from the imputation model; only baseline clinical covariates with incomplete observations underwent imputation (scikit-learn IterativeImputer, max_iter = 50, random_state = 42, sample_posterior = True). Given the relatively limited sample size, FIGO stage and treatment line were modeled as ordinal integer covariates to preserve statistical efficiency and avoid model overparameterization.

(iii) Honesty and Cross-fitting: Honesty was enforced through the subsampling mechanism built into the GRF framework: for each tree, data were randomly partitioned into splitting and estimation subsets, where the former determined tree structure and the latter estimated treatment effect sizes within the identified leaf boundaries. This subsampling-based honesty procedure reduces overfitting and supports valid asymptotic inference. Confidence intervals were derived using the asymptotic variance estimator implemented in the GRF framework. Asymptotic variance estimation was enabled via inference = True to support valid confidence interval computation. To assess the stability of the concordance analysis, bootstrap resampling (1,000 iterations) was applied to derive the 95% confidence interval of the observed concordance rate.

(III) Mapping the Statistical Relationship:

The resulting individualized treatment response difference estimates and their 95% confidence intervals were plotted as a continuous function of OVS, providing a descriptive summary of how estimated treatment response patterns vary across the observed OVS range.

### 2.4 External reproducibility analysis

To assess the directional reproducibility of the observed OVS-associated treatment heterogeneity pattern, a prespecified validation analysis was conducted in an independent real-world cohort of 58 eligible patients recruited from two tertiary academic centers (Xiangya Hospital and the Second Xiangya Hospital, Central South University), independent of the primary cohort at all stages of OVS construction, model specification, and effect estimation. Harmonization procedures were applied to align variable definitions, inclusion criteria, treatment classification, and outcome ascertainment. The predefined OVS framework, component definitions, threshold specifications, and covariate-adjusted modeling strategy were applied without modification or recalibration. Given the limited sample size, the external validation was designed to evaluate qualitative concordance of treatment effect directionality across OVS-defined strata rather than to provide a fully powered independent replication; results are interpreted as assessments of directional consistency rather than confirmatory evidence.

### 2.5 Robustness and validation analyses

To evaluate the robustness of the study findings under alternative assumptions, data handling strategies, and model specifications, we conducted a pre-specified set of complementary analyses. These analyses were designed to assess the stability of the estimated treatment effect heterogeneity rather than to formally validate a predictive model.

#### 2.5.1 Robustness to missing data assumptions and heterogeneous treatment effect estimation

- Complete-Case Analysis: CCA was performed by restricting the dataset to patients with fully observed baseline covariates. Consistency between CCA and primary MI-based estimates was evaluated to assess the influence of missing data handling on the primary findings.
- Subgroup Consistency Analyses: The analysis was repeated across four predefined subgroups: (i) patients with at least one available molecular marker result (BRCA or HRD); (ii) patients with both BRCA and HRD status unavailable; (iii) the platinum-sensitive subcohort; and (iv) patients with advanced-stage disease (FIGO stage III–IV). Subgroups (i) and (ii) were mutually complementary and designed to evaluate whether the OVS-mediated treatment interaction remained consistent across different levels of molecular data availability.
- Threshold Invariance: The treatment–OVS interaction was re-evaluated using alternative OVS dichotomization thresholds (OVS ≥ 1 and OVS ≥ 3) to assess whether the qualitative direction of treatment effect heterogeneity remained stable across different definitions of physiological vulnerability.

#### 2.5.2 Sensitivity to unmeasured confounding

E-values were calculated from the observed interaction hazard ratios using standard methodology for ratio effect measures, representing the minimum strength of association that an unmeasured confounder would need with both treatment assignment and PFS to fully explain the observed interaction, conditional on measured covariates. E-values were computed for both the point estimate and the upper confidence interval bound (the bound closest to the null value of 1.0).

#### 2.5.3 Internal consistency assessment of the OVS construct

Internal consistency analyses were performed to evaluate the coherence and robustness of the OVS as a composite measure of baseline physiological vulnerability. Because OVS was developed as an effect-modifying construct rather than a prognostic prediction model, these analyses assessed construct-level consistency rather than predictive accuracy.

- Outcome ranking consistency: Harrell’s C-index for PFS was calculated descriptively to assess whether OVS demonstrated a non-random association with survival outcome ordering.
- Construct coherence across vulnerability strata: OVS-stratified Kaplan–Meier survival curves were examined across all four OVS categories (0, 1, 2, and 3) to assess whether progressively higher OVS values were associated with a monotonic gradient of worsening PFS outcomes.
- Alternative specification analysis: To assess the robustness of the OVS framework to alternative organ-level vulnerability definitions, two stricter organ-level criteria were evaluated alongside the primary definition (≥ 1 abnormal indicator per organ system classifies that organ as vulnerable): (i) ≥ 2 abnormal indicators required per organ system; and (ii) all indicators within an organ system must exceed predefined thresholds. Under each organ-level framework, the total OVS dichotomization threshold was additionally varied (OVS ≥ 1 and OVS ≥ 3, in addition to the primary OVS ≥ 2) to assess whether the qualitative direction of the treatment–OVS interaction remained stable across varying definitions of high physiological vulnerability.

#### 2.5.4 Specificity of OVS as a composite effect modifier

To assess whether the observed treatment effect heterogeneity was attributable to the composite physiological construct rather than any single biomarker alone, a systematic substitution analysis was conducted. OVS was sequentially replaced by candidate individual biomarkers previously associated with PFS in prior studies within the same IPTW-weighted interaction modeling framework (13). Candidate biomarkers included all individual indicators comprising the OVS construct (hemoglobin, platelet count, absolute neutrophil count, albumin, total bilirubin, AST, creatinine, and uric acid), supplemented by additional biomarkers previously associated with PFS in prior studies (fibrinogen, total bile acids, and thrombin time) and key clinical baseline variables (age and FIGO stage). Each biomarker was dichotomized using the same clinically prespecified thresholds applied in OVS construction where applicable; for biomarkers with established clinical reference standards, prespecified thresholds were applied: fibrinogen >4.0 g/L, total bile acids >10.0 μmol/L, and thrombin time >21.0 seconds, consistent with WS/T 404–2012 reference intervals and Chinese coagulation assessment standards. For age and FIGO stage, which lack universal clinical dichotomization thresholds, the sample median was used as the cutoff.

All computational procedures were performed in Python 3.9. The analytical toolkit included lifelines for survival analysis and Cox modeling; scikit-learn for preprocessing and multiple imputation (IterativeImputer); pandas and numpy for data management; scipy for statistical testing; statsmodels for regression diagnostics; and econml for Causal Forest-based CATE estimation. Visualization used matplotlib and seaborn. Source code is publicly available (see **Data Availability Statement**).

## 3 Study results

### 3.1 Baseline characteristics of the study population and distribution of organ vulnerability

A total of 604 patients with ovarian cancer receiving olaparib or niraparib maintenance therapy were included in the study cohort. The mean age was 55.99 years. BRCA mutation testing was available in 280 patients (46.36%), whereas HRD testing was available in 74 patients (12.25%), reflecting incomplete molecular profiling in routine clinical practice. Among patients with available BRCA testing, 135 (48.21%) harbored BRCA mutations. Detailed baseline characteristics are summarized in **Table 1**.

**Table 1:** Baseline characteristics of the study cohort (n = 604).

Characteristics (category)	Statistics
Demographics	
Age (years)	55.99 ± 8.26
Height (m)	1.56 ± 0.05
Weight (kg)	55.12 ± 8.63
BMI (kg/m²)	22.63 ± 3.30
Tumor characteristics	
Pathological type	
Serous carcinoma	538 (89.07%)
Mucinous carcinoma	46 (7.62%)
Clear cell carcinoma	8 (1.32%)
Endometrioid carcinoma	12 (1.99%)
FIGO stage	
Stage I	8 (1.33%)
Stage II	22 (3.66%)
Stage III	450 (74.88%)
Stage IV	121 (20.13%)
Missing	3
Metastasis status	
No metastasis	48 (7.95%)
Organ metastasis	151 (25.00%)
Peritoneal metastasis	405 (67.05%)
BRCA mutation status	
Wild-type	145 (51.79%)
Mutated	135 (48.21%)
Missing	324
HRD status	
Negative	24 (32.43%)
Positive	50 (67.57%)
Missing	530
Differentiation grade	
Well-differentiated	407 (87.15%)
Moderately/Poorly differentiated	60 (12.85%)
Missing	137
Treatment regimens	
Pre-PARP treatment regimen	
Taxane-Platinum	273 (45.20%)
Taxane-Platinum + Combination	292 (48.34%)
Others	39 (6.46%)
Maintenance drug	
Olaparib	445 (73.68%)
Niraparib	159 (26.32%)
Platinum sensitivity status	
Sensitive	573 (98.62%)
Resistant	8 (1.38%)
Missing	23
Laboratory examinations	
Absolute neutrophil count (×10⁹/L)	8.036 ± 6.04
Absolute lymphocyte count (×10⁹/L)	1.207 ± 0.57
Thrombin time (s)	16.20 ± 1.44
Hemoglobin (g/L)	111.1 ± 17.68
Platelet count (×10⁹/L)	236.3 ± 115.4
AST (U/L)	30.28 ± 21.15
Urea (mmol/L)	4.67 ± 1.88
Uric acid (μmol/L)	282.00 ± 91.03
Creatinine (μmol/L)	59.90 ± 17.17
Total bilirubin (μmol/L)	12.28 ± 5.44
Follow-up information	
PFS (months)	25.10 ± 14.58
Median follow-up time (months)	36.5 (Follow-up period: 2019-01–15 to 2024-06-30)

Continuous variables are presented as mean ± standard deviation (SD), and categorical variables as number (percentage).

**Figure 3:**
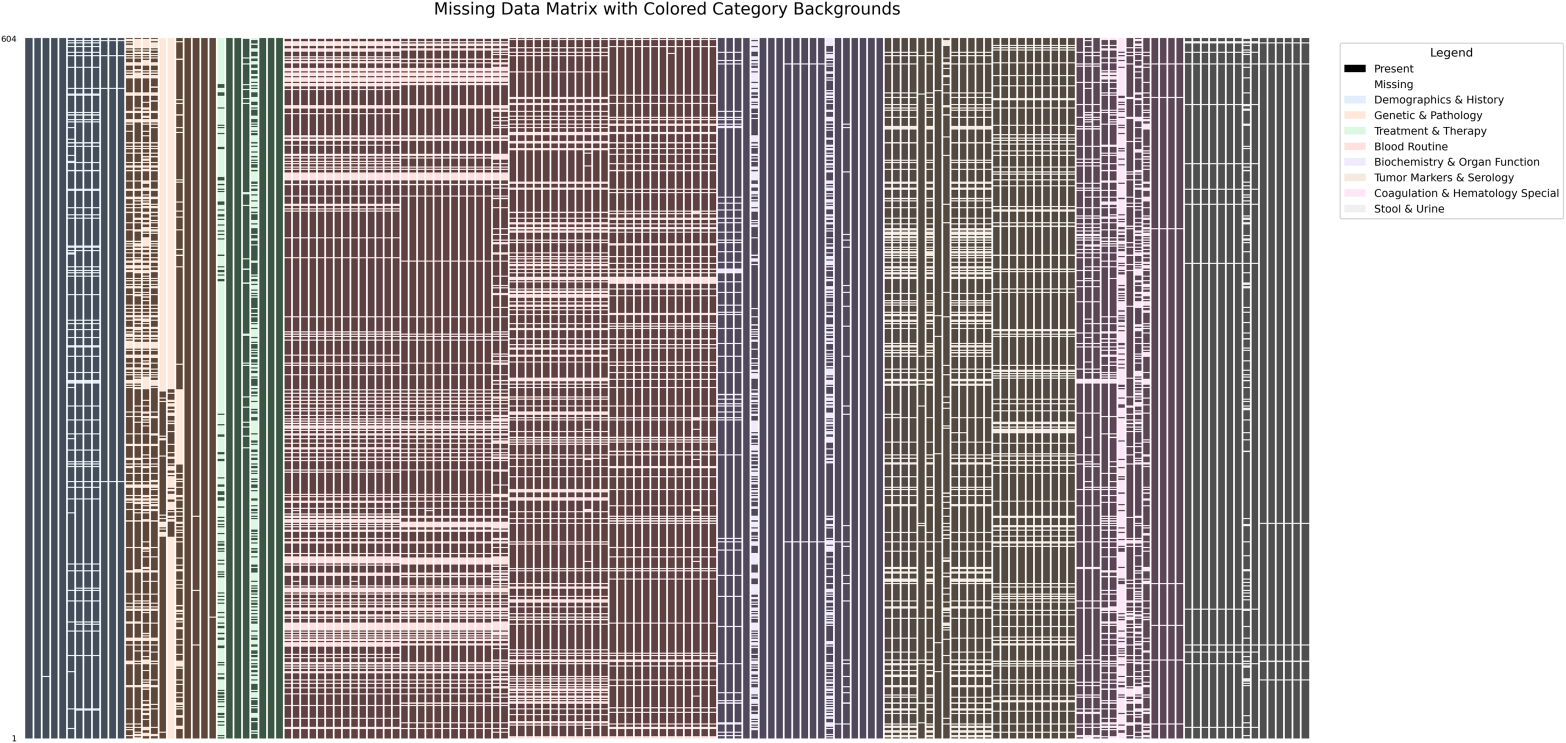
Heatmap of missing data patterns across 604 patients, stratified by variable category.

To characterize data completeness across the study cohort, a missing data matrix plot was constructed for all baseline variables (**Figure 3**). Black cells indicate observed values and white cells indicate missing values. Most clinical and laboratory variables demonstrated high completeness, particularly key organ function indicators used in the OVS framework, including hemoglobin, AST, and creatinine.

**Table 2** summarizes the distribution of OVS categories and baseline characteristics stratified by physiological vulnerability status. Among the 604 patients, 32.6% (n = 197) had an OVS of 0, 46.7% (n = 282) had an OVS of 1, 17.4% (n = 105) had an OVS of 2, and 3.3% (n = 20) had an OVS of 3. Overall, 79.3% (n = 479) of patients were classified as low vulnerability (OVS 0–1), whereas 20.7% (n = 125) were classified as high vulnerability (OVS ≥2).

**Table 2:** Baseline characteristics stratified by organ vulnerability status.

Characteristic	Total (N = 604)	Low vulnerability (score = 0–1; n = 479)	High vulnerability (score ≥ 2; n = 125)
OVS, n (%)			
0	197 (32.6%)	197 (41.1%)	0 (0.0%)
1	282 (46.7%)	282 (58.9%)	0 (0.0%)
2	105 (17.4%)	0 (0.0%)	105 (84.0%)
3	20 (3.3%)	0 (0.0%)	20 (16.0%)
Age, mean (SD), years	56.0 (8.3)	55.5 (8.1)	58.0 (8.6)
Maintenance drug, n (%)			
Olaparib	445 (73.7%)	349 (72.9%)	96 (76.8%)
Niraparib	159 (26.3%)	130 (27.1%)	29 (23.2%)
BRCA status, n (%)			
Mutated	135 (22.4%)	107 (22.3%)	28 (22.4%)
Wild-type	145 (24.0%)	122 (25.5%)	23 (18.4%)
Unknown	324 (53.6%)	250 (52.2%)	74 (59.2)
HRD status, n (%)			
HRD (+)	50 (8.2%)	37 (7.7%)	13 (10.4%)
HRD (–)	24 (4.0%)	22 (4.6%)	2 (1.6%)
Unknown	530 (87.8%)	420 (87.7%)	110 (88.0%)

Compared with the low-vulnerability group, patients in the high-vulnerability group were slightly older and had a modestly lower proportion of niraparib use. Molecular testing patterns were generally similar across OVS strata, although the proportion of patients with unknown BRCA/HRD status remained high in both groups, reflecting incomplete molecular profiling in real-world practice.

### 3.2 Methodological benchmarking using BRCA Status and PFS

**Figure 4:**
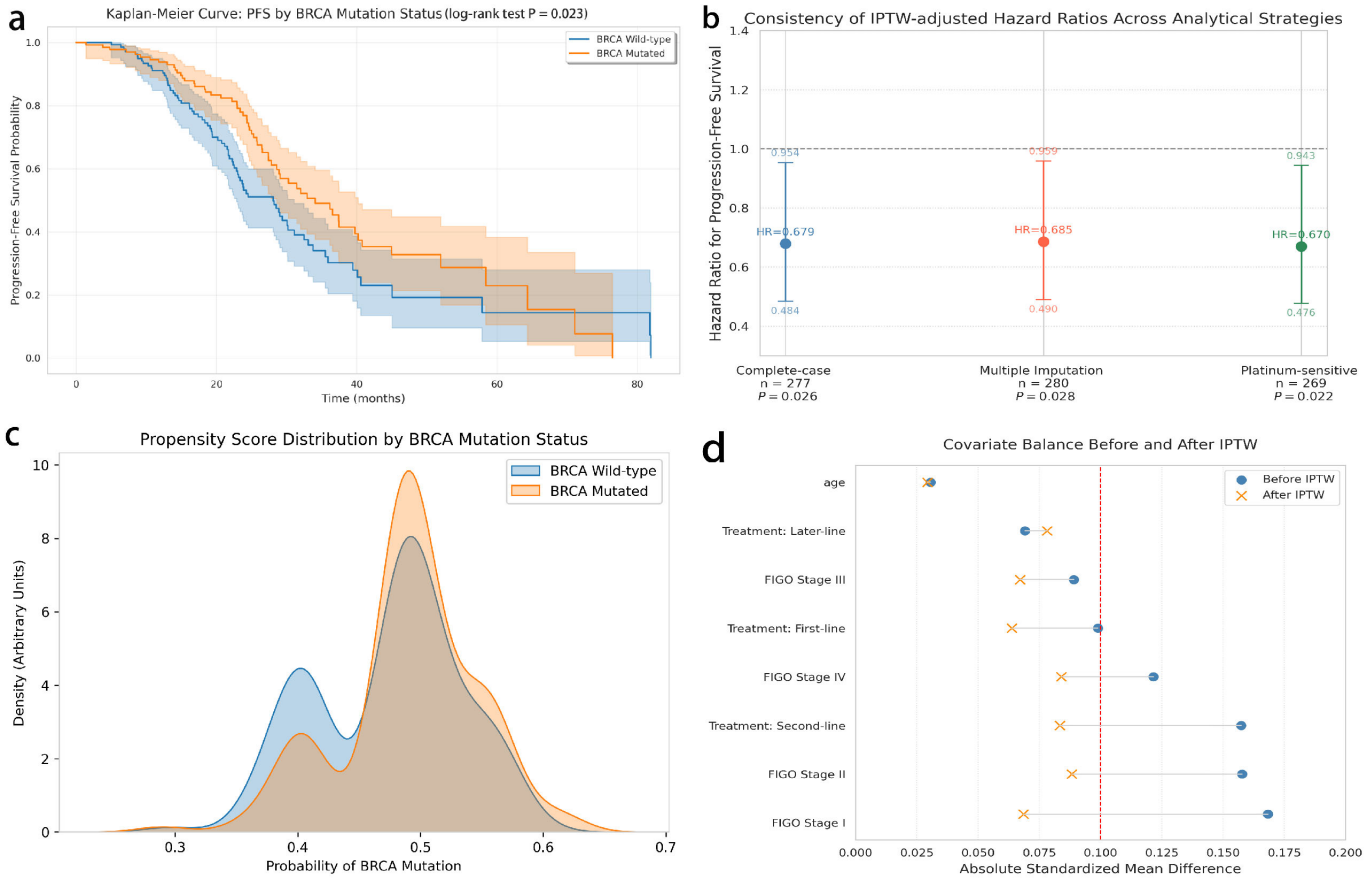
Benchmark validation of the BRCA–PFS relationship. **(a)** Kaplan–Meier curves of PFS stratified by BRCA mutation status. Shaded areas represent 95% confidence intervals. **(b)** IPTW-adjusted hazard ratios for BRCA mutation across different analytical strategies. Blue: CCA; red: MI; green: platinum-sensitive subgroup (MI-based). **(c)** Distribution of propensity scores by BRCA status, demonstrating adequate overlap between exposure groups. **(d)** Covariate balance before and after IPTW, assessed using SMDs. All covariates achieved absolute SMD < 0.1 after weighting, indicating adequate covariate balance.

Among 280 patients with available BRCA mutation status, we first conducted an unadjusted Kaplan–Meier analysis comparing PFS between BRCA-mutated and BRCA wild-type patients. Consistent with previously reported clinical observations, BRCA-mutated patients exhibited longer PFS than wild-type patients (log-rank test P = 0.023; **Figure 4a**).

This analysis was designed as a methodological benchmark rather than an independent inferential objective, aiming to evaluate whether the proposed analytical framework could recover a well-recognized biological association under real-world data conditions. To reduce imbalance in measured baseline characteristics between BRCA-mutated and wild-type patients, inverse probability weighting based on propensity scores was applied using age, FIGO stage, and number of prior platinum-based chemotherapy lines.

In the primary analysis, MI was used to address limited missingness in baseline covariates. The IPTW-weighted Cox model yielded a hazard ratio (HR) of 0.685 (95% CI: 0.490–0.959; P = 0.028), indicating longer PFS in BRCA-mutated patients. CCA, restricted to patients with fully observed baseline covariates (n = 277), yielded highly consistent estimates (HR = 0.679, 95% CI: 0.484–0.954; P = 0.026), suggesting that the minimal degree of missingness had negligible influence on the estimated association. To further evaluate the stability of the findings, the analysis was repeated in the platinum-sensitive subgroup (n = 269). The estimated effect remained consistent in direction and magnitude (HR = 0.670, 95% CI: 0.476–0.943; P = 0.022), supporting the robustness of the observed association across clinically relevant subpopulations.

Covariate balance diagnostics demonstrated that IPTW substantially improved baseline comparability between BRCA-mutated and wild-type groups. All absolute standardized mean differences were reduced to below 0.1 after weighting (**Figure 4d**; **Supplementary Table 1**), indicating adequate covariate balance. In addition, the distribution of propensity scores showed substantial overlap between exposure groups (**Figure 4c**), supporting the positivity assumption.

In summary, this benchmark analysis shows that the proposed analytical framework can recover the established association between BRCA mutation status and PFS—as documented in prior clinical trials (1, 2, 4, 5)—across multiple analytical strategies. The consistency of effect estimates across CCA, MI, and subgroup analyses, together with satisfactory covariate balance and propensity score overlap, supports the methodological consistency and operational reliability of the analytical framework for subsequent exploratory analyses.

### 3.3 Organ vulnerability as a modifier of the relative efficacy of PARP inhibitors

This analysis was conducted in the full cohort (n = 604). To evaluate whether baseline physiological vulnerability modified the relative effectiveness of PARP inhibitors on PFS, we fitted an IPTW-weighted multivariable Cox proportional hazards model incorporating a treatment-by-OVS interaction term, combined with G-computation-based causal estimation. The interaction analysis suggested significant treatment effect heterogeneity across OVS levels (interaction HR = 0.488, 95% CI: 0.293–0.813; P = 0.006; **Table 3**). Specifically, the relative PFS benefit associated with olaparib, compared with niraparib, became more pronounced with increasing OVS, suggesting that baseline physiological vulnerability modified the relative effectiveness of the two PARP inhibitors.

**Table 3:** IPTW-weighted Cox proportional hazards model with treatment-by-OVS interaction.

Variable	HR (95% CI)	P value
Age (per year)	1.035 (0.926–1.156)	0.546
Stage	1.269 (1.063–1.515)	0.008
Treatment line	1.791 (1.518–2.113)	< 0.001
Treatment (Olaparib vs Niraparib)	0.744 (0.541−1.022)	0.068
OVS	1.062 (0.792–1.426)	0.687
Treatment × OVS interaction	**0.488 (0.293–0.813)**	**0.006**

The bolded HR = 0.488 (95% CI: 0.293–0.813; P = 0.006) of treatment-by-OVS interaction indicates that the relative progression-free survival benefit associated with olaparib, compared with niraparib, became more pronounced with increasing OVS levels. The OVS was included in the model solely to estimate the interaction term and its main effect; it was not included in the confounder adjustment set. The main effect of OVS in this model should not be interpreted as a prognostic estimate.

**Figure 5:**
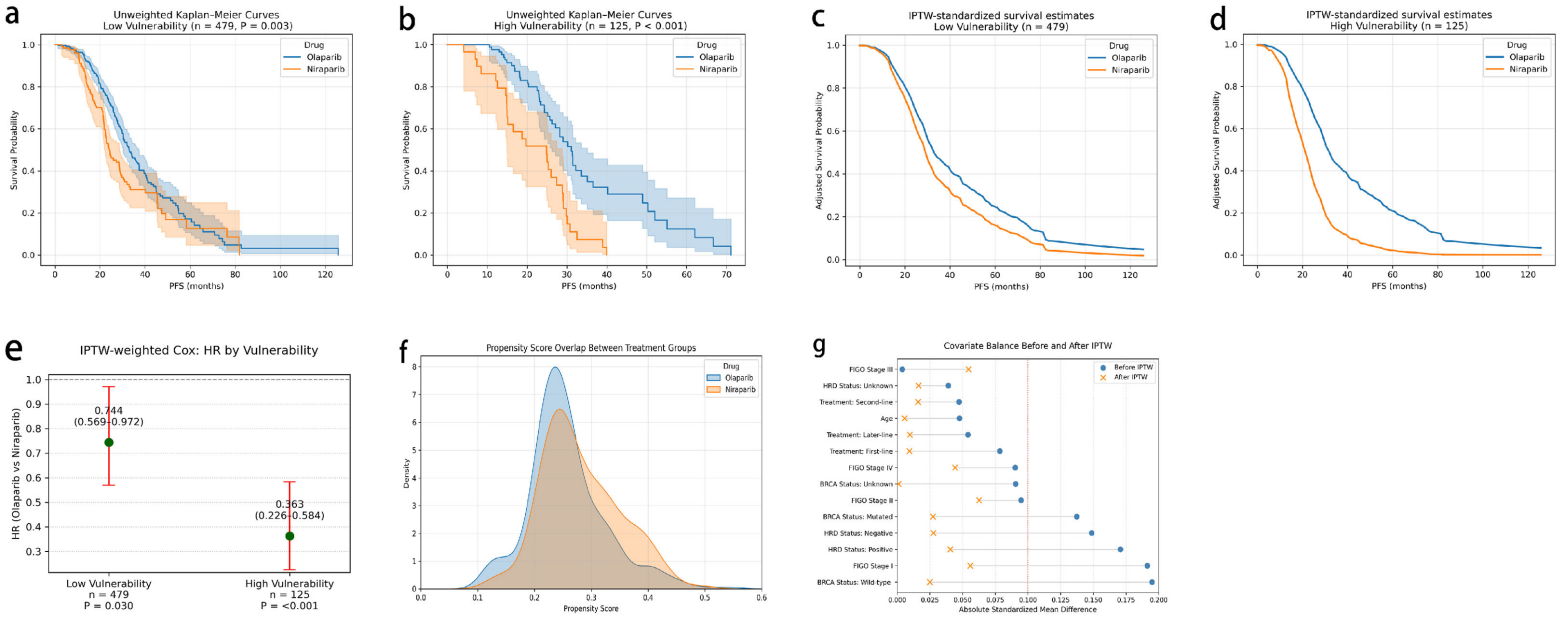
OVS-associated heterogeneity in the relative effectiveness of olaparib versus niraparib. **(a, b)** Kaplan–Meier curves of PFS stratified by OVS category and treatment group. **(a)** Low-vulnerability subgroup (OVS 0–1, n = 479); **(b)** high-vulnerability subgroup (OVS 2–3, n = 125). Shaded areas represent 95% confidence intervals. (c–d) IPTW-standardized survival estimates derived from weighted Cox proportional hazards models. **(c)** Low-vulnerability subgroup; **(d)** high-vulnerability subgroup. **(e)** Hazard ratio estimates comparing olaparib versus niraparib across OVS strata. **(f)** Propensity score distributions by treatment group, demonstrating substantial overlap between olaparib and niraparib recipients. **(g)** SMDs before and after IPTW adjustment. All post-weighting covariates achieved absolute SMD < 0.1, indicating adequate balance of measured baseline characteristics.

Kaplan–Meier analyses and IPTW-standardized survival estimates demonstrated distinct PFS patterns between olaparib and niraparib across OVS-defined vulnerability strata (**Figure 5**). Among patients with low organ vulnerability (score 0–1, n = 479), olaparib was associated with a modest PFS benefit compared with niraparib (HR = 0.744, 95% CI: 0.569–0.972; P = 0.030; **Figures 5a, e**). This pattern was further reflected in the IPTW-standardized survival curves, which showed a moderate separation between treatment groups over time (**Figure 5c**).

In patients with high organ vulnerability (score 2–3, n = 125), the relative PFS benefit associated with olaparib was substantially greater (HR = 0.363, 95% CI: 0.226–0.584; P < 0.001; **Figures 5b, e**). Correspondingly, the IPTW-standardized survival curves demonstrated earlier and more pronounced separation between treatment groups in the high-vulnerability subgroup (**Figure 5d**), with the niraparib group exhibiting a steeper decline during early follow-up.

Although these observations may reflect differences in treatment tolerance or physiological reserve in vulnerable patients, they should be interpreted within the observational and model-based nature of the analysis. Propensity score distributions demonstrated substantial overlap between treatment groups (**Figure 5f**), supporting the plausibility of the positivity assumption. In addition, post-weighting covariate balance diagnostics showed that all SMDs were reduced to below 0.1 after IPTW adjustment (**Figure 5g**; **Supplementary Table 2**), indicating adequate balance of measured baseline covariates. The effective sample size after weighting was 562.3 (ESS/N ratio = 0.931), indicating well-concentrated stabilized weights without substantial variance inflation. This finding is consistent with the observed propensity score overlap and the moderate degree of pre-weighting covariate imbalance, reflecting that treatment group differences in measured baseline characteristics were amenable to adjustment without requiring extreme reweighting.

The consistency of the observed pattern across both the interaction model and the IPTW-standardized survival estimates supports the internal coherence of the OVS-associated treatment heterogeneity signal within the observed dataset.

### 3.4 Exploratory analysis of treatment heterogeneity via causal forest

**Figure 6:**
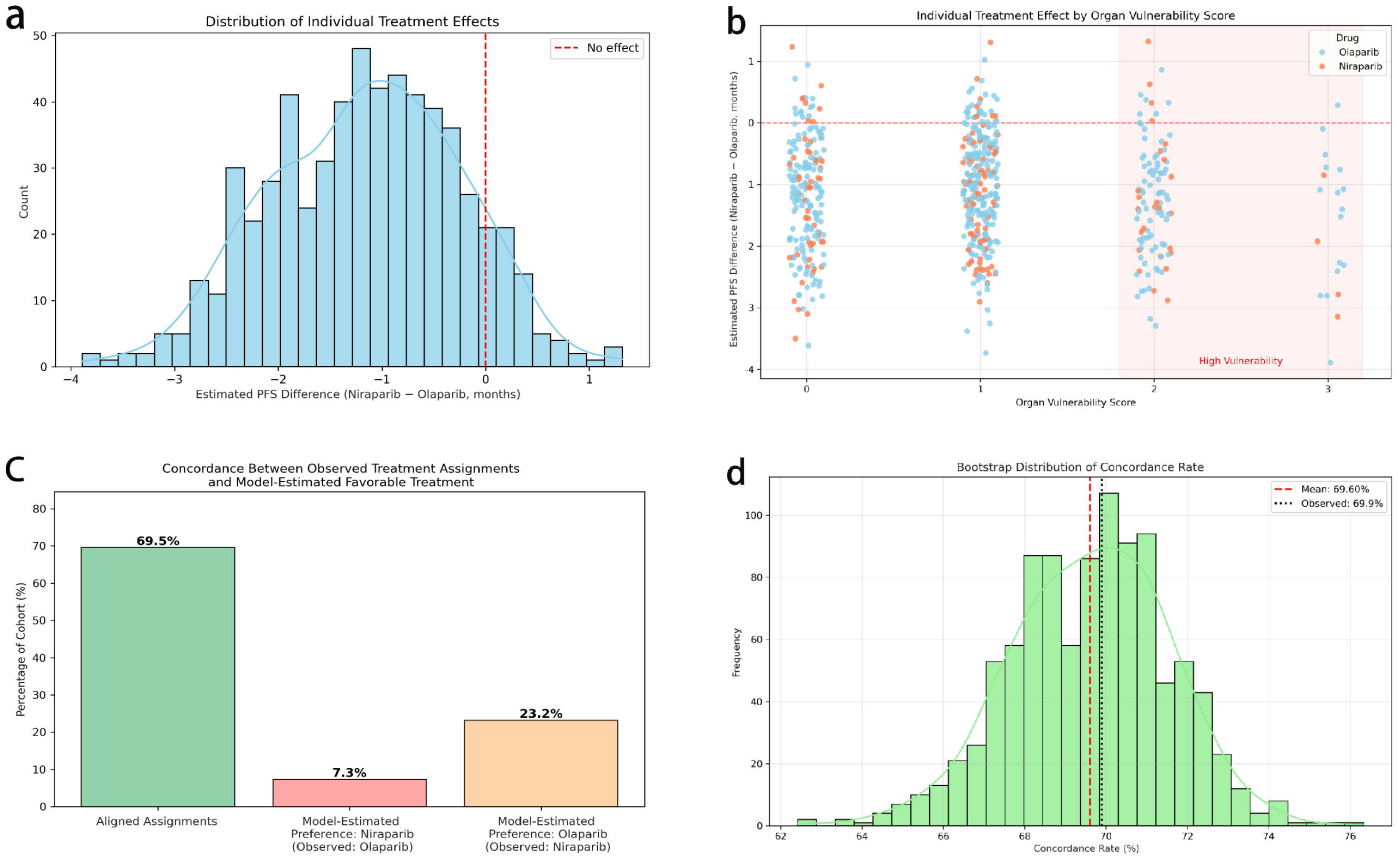
**(a)** Distribution of estimated individual treatment response differences from the causal forest model. Negative values indicate relatively more favorable estimated PFS outcomes under olaparib; these estimates reflect differential PFS duration under observed treatment conditions and should be interpreted as exploratory rather than causal. **(b)** Estimated treatment response differences across OVS levels. **(c)** Concordance between observed treatment assignments and model-estimated favorable treatment. **(d)** Bootstrap distribution of the concordance rate across 1,000 resampling iterations.

The distribution of estimated individual treatment response differences across the full cohort was left-skewed, with both the median and mean located below zero (**Figure 6a**), indicating that olaparib was generally associated with more favorable estimated PFS outcomes than niraparib at the population level. Nevertheless, a subset of patients with positive estimated response differences remained observable in the right tail of the distribution, suggesting potential heterogeneity in relative treatment response.

When stratified by OVS, the distribution of estimated treatment response differences progressively shifted downward with increasing vulnerability levels (**Figure 6b**), indicating that the relative estimated benefit of olaparib became more pronounced in patients with higher physiological vulnerability. Among low-vulnerability patients (OVS 0–1), a substantial proportion of individuals exhibited positive estimated response differences, suggesting that niraparib may remain comparatively favorable in a subset of patients. In contrast, most high-vulnerability patients (OVS ≥ 2) demonstrated negative estimated response differences, consistent with a stronger relative association between olaparib and improved PFS in this subgroup. These findings were directionally consistent with the stratified estimates obtained from the G-computation analysis in Section 3.3.

To descriptively assess the concordance between observed prescribing patterns and model-estimated treatment preference, we conducted an exploratory alignment analysis (**Figure 6c**). For each patient, the treatment associated with the more favorable estimated CATE was compared with the actual observed treatment assignment. Within the observed cohort, 69.5% of treatment assignments were concordant with the model-estimated favorable treatment category. Among discordant cases, 7.3% of patients received olaparib despite a relatively more favorable estimated effect for niraparib, whereas 23.2% received niraparib despite a relatively more favorable estimated effect for olaparib.

This analysis should be interpreted strictly as an exploratory comparison between observed prescribing patterns and model-estimated treatment heterogeneity, rather than as a clinical decision-making metric. The estimated concordance rates represent a theoretical pattern within the observed data structure and depend on the measured covariates and identifying assumptions of the causal framework. Real-world treatment selection may additionally be influenced by factors not captured in the present dataset, including physician preference, institutional practice patterns, treatment accessibility, and patient-specific considerations.

To assess the internal stability of the alignment estimates, a bootstrap resampling analysis with 1,000 replications was performed. The bootstrap mean concordance rate was 69.6% (95% CI: 65.9%–73.3%), with an approximately symmetric distribution (**Figure 6d**), suggesting that the estimated alignment pattern was reasonably stable within the present dataset. The directional pattern of OVS-associated treatment heterogeneity observed in the Cox interaction analysis was qualitatively consistent with the individual-level response variation identified by the exploratory causal forest analysis, providing cross-model support for the observed heterogeneity signal.

### 3.5 External consistency assessment of OVS-associated treatment heterogeneity

Baseline characteristics of the external validation cohort are summarized in **Supplementary Table 3**, and detailed interaction model results are provided in **Supplementary Table 4** and **Supplementary Figure 1**. In the independent external cohort (n = 58), OVS-stratified treatment effect estimates were obtained using the same predefined analytical framework, covariate adjustment strategy, and OVS threshold specifications as the primary analysis, without any recalibration or re-estimation within the external dataset.

Among patients with lower physiological vulnerability, the estimated association between olaparib and progression-free survival was comparatively modest and did not reach statistical significance (HR = 0.857, 95% CI: 0.628–1.170). Among patients with higher physiological vulnerability, a stronger estimated association favoring olaparib was observed (HR = 0.466, 95% CI: 0.243–0.892). The treatment-by-OVS interaction term demonstrated a directionally concordant effect estimate relative to the primary cohort, although statistical significance was not reached in the external dataset (interaction HR = 0.544, P = 0.106).

Several observations warrant cautious interpretation. The attenuation of the estimated treatment effect in the low-vulnerability stratum—and its loss of statistical significance relative to the primary cohort—most plausibly reflects the substantially reduced statistical power available in the smaller external sample rather than a fundamentally discordant effect pattern, though this cannot be formally confirmed. The interaction term, while directionally consistent, similarly did not achieve statistical significance, which is expected given the limited sample size.

Overall, these results are interpreted as preliminary assessments of directional reproducibility rather than formal independent replication. The qualitative concordance of the high-vulnerability stratum estimate across independent cohorts provides limited supportive evidence for the OVS-associated treatment heterogeneity pattern, while the reduced precision of all estimates in the external cohort underscores the need for prospective evaluation in larger, multicenter datasets before any clinical application of this framework can be considered.

### 3.6 Sensitivity and robustness analysis

#### 3.6.1 Robust validation of organ vulnerability and its interaction with PARP inhibitor efficacy

To evaluate the robustness of the primary findings under alternative analytical assumptions, population restrictions, and OVS definitions, a series of complementary robustness analyses were performed (**Table 4**). Across most analytical scenarios, the interaction between OVS and PARP inhibitor treatment remained directionally consistent with the primary analysis, supporting the stability of the observed treatment effect heterogeneity.

**Table 4:** Robustness analyses of OVS-associated treatment effect heterogeneity across alternative analytical scenarios.

Analysis	Interaction HR (P)	HR in high OVS (95% CI)
Primary analysis by CCA (601)	0.489(0.005)	0.362 (0.228–0.587)
Primary analysis by MI (604)	0.488(0.006)	0.363 (0.226–0.584)
BRCA/HRD any known (299)	0.490(0.048)	0.449 (0.218–0.925)
BRCA/HRD both unknown (305)	0.499(0.009)	0.371 (0.210–0.658)
Platinum-sensitive (573)	0.489(0.015)	0.410 (0.250–0.671)
Only III–IV stage (571)	0.488(0.005)	0.404 (0.266–0.615)
OVS ≥ 1 as High vulnerability (604)	0.782(0.399)	0.604 (0.459–0.794)
OVS ≥ 2 as High vulnerability (604)	0.488(0.006)	0.363 (0.226–0.584)
OVS ≥ 3 as High vulnerability (604)	0.383(0.045)	0.256 (0.073–0.894)

Interaction HRs were estimated from IPTW-weighted Cox models incorporating treatment-by-OVS interaction terms. HRs <1 in the high-OVS subgroup indicate relative benefit of olaparib versus niraparib.

Comparison between CCA and MI demonstrated highly consistent results. In both analyses, patients with high physiological vulnerability exhibited relatively more favorable PFS outcomes with olaparib versus niraparib, and the estimated interaction effect remained statistically significant. The similarity between CCA and MI estimates suggests that limited missingness in baseline covariates had minimal influence on the primary findings.

To further assess robustness to molecular data availability, the cohort was divided into two complementary subgroups: (i) patients with at least one known BRCA or HRD result (n = 299), and (ii) patients with both BRCA and HRD status unavailable (n = 305). Directionally consistent interaction patterns were observed in both subgroups. Notably, the interaction remained statistically significant in the molecularly uncharacterized subgroup, suggesting that physiological vulnerability may provide complementary stratification information in settings where molecular profiling is unavailable. In the subgroup with available molecular information, a similar effect pattern was observed, potentially reflecting the stronger prognostic contribution of molecular characteristics in this subgroup.

Additional subgroup analyses were conducted in platinum-sensitive patients (n = 573) and in patients with advanced-stage disease (FIGO stage III–IV, n = 571). In both scenarios, the estimated interaction effects remained directionally consistent with the primary analysis, indicating that the observed OVS-associated treatment heterogeneity was not primarily driven by platinum sensitivity distribution or disease stage composition.

Sensitivity analyses using alternative OVS dichotomization thresholds demonstrated that the estimated treatment interaction varied according to the degree of physiological vulnerability enrichment. When high vulnerability was defined as OVS ≥ 1, the interaction effect was attenuated and no longer statistically significant (interaction HR = 0.782, P = 0.399), likely reflecting increased heterogeneity introduced by inclusion of patients with relatively mild physiological impairment. In contrast, stronger interaction effects were observed under stricter vulnerability definitions. The prespecified OVS ≥ 2 threshold demonstrated a significant interaction effect (interaction HR = 0.488, P = 0.006). Under the most restrictive threshold (OVS ≥ 3), the estimated interaction HR was 0.383 (P = 0.045); however, given the very small number of patients meeting this criterion, confidence intervals were substantially wider and this estimate should be interpreted with considerable caution. Overall, the direction of treatment heterogeneity remained consistent across alternative OVS thresholds.

Interaction HRs were estimated from IPTW-weighted Cox models incorporating treatment-by-OVS interaction terms. HRs <1 in the high-OVS subgroup indicate relative benefit of olaparib versus niraparib.

#### 3.6.2 Sensitivity to unmeasured confounding: E-value analysis

To assess the robustness of the observed treatment–OVS interaction to potential unmeasured confounding, an E-value analysis was performed for the primary interaction estimate. The calculated E-value was 3.513, indicating that an unmeasured confounder would need to be associated with both treatment assignment and progression-free survival by an association of at least 3.513 on the risk ratio scale, above and beyond the measured covariates, to fully explain away the observed interaction effect.

Because the primary estimand involved a treatment-by-OVS interaction term, such an unmeasured factor would also need to induce substantial joint imbalance related to both treatment allocation and physiological vulnerability. While an E-value of 3.513 does not preclude residual confounding—particularly from factors such as physician prescribing preference or performance status that may plausibly exert moderate-to-strong associations with both treatment selection and survival—it quantifies the minimum strength of unmeasured confounding that would be required to fully explain the observed interaction, providing a reference benchmark for interpreting the robustness of the finding.

#### 3.6.3 Internal consistency assessment of the OVS construct

As expected for a physiological effect-modifying construct rather than a dedicated prognostic prediction model, the OVS demonstrated modest discrimination for PFS ranking (Harrell’s C-index: 0.622). OVS-stratified Kaplan–Meier analysis demonstrated a directional gradient in PFS outcomes across increasing OVS categories (log-rank P = 0.011; **Supplementary Figure 2**), with patients in the OVS 0 stratum exhibiting the longest estimated PFS. While the magnitude of survival differences across intermediate OVS strata was modest and the curve for OVS 3 should be interpreted cautiously given the small stratum size (n = 20), the overall directional pattern is consistent with the construct’s intended representation of increasing physiological vulnerability. Importantly, the modest prognostic discrimination observed for OVS is not inconsistent with its role as an effect modifier: a variable may exhibit limited independent prognostic value while meaningfully modifying the relative effectiveness of competing treatments, as the two properties reflect distinct statistical estimands.

Construct robustness was further assessed using progressively stricter organ-level definitions, including requiring at least two or all indicators within an organ system to exceed predefined thresholds. Under the strictest specification, OVS values became highly compressed, necessitating dichotomization at OVS ≥ 1. Despite these increasingly restrictive definitions, the treatment–OVS interaction remained directionally consistent across alternative specifications and appeared more pronounced under the strictest construct definition, potentially reflecting enrichment of patients with more severe physiological vulnerability. Similar findings were observed across alternative OVS dichotomization thresholds (**Supplementary Table 5**), supporting the internal robustness and construct consistency of the OVS framework.

### 3.6.4 Specificity of the composite OVS framework

To evaluate whether the observed treatment–OVS interaction could be explained by any single clinical or laboratory variable alone, the composite OVS was sequentially replaced with individual baseline variables previously associated with PFS in ovarian cancer studies, including all individual indicators comprising the OVS construct (hemoglobin, platelet count, absolute neutrophil count, albumin, total bilirubin, AST, creatinine, and uric acid), supplemented by additional biomarkers previously associated with PFS in prior studies (fibrinogen, total bile acids, and thrombin time) and key clinical variables (age and FIGO stage) (13). Separate IPTW-weighted Cox models incorporating “drug × variable” interaction terms were then constructed for each candidate variable.

None of the individual variables demonstrated interaction effects comparable to those observed for the composite OVS framework (**Supplementary Table 6**). In contrast, the OVS consistently retained a statistically significant treatment interaction signal across model specifications. These findings support the specificity of the composite OVS construct and suggest that the observed treatment heterogeneity may reflect integrated multi-system physiological vulnerability rather than the isolated effect of any single biomarker or clinical characteristic.

## 4 Discussion

This study investigated potential sources of treatment heterogeneity in PARP inhibitor efficacy within a causal inference framework. In the primary exploratory analysis, baseline OVS was associated with differences in the relative effectiveness patterns of olaparib and niraparib. Notably, the presence of a significant treatment-by-OVS interaction (P = 0.006), despite the absence of a significant main effect for OVS itself, supports the interpretation of OVS primarily as an effect modifier rather than an independent prognostic factor. This pattern is consistent with HTE, whereby baseline physiological vulnerability may be associated with differences in the relative effectiveness of PARP inhibitors across patient subgroups (18). Importantly, the present analysis characterizes relative treatment patterns within the context of real-world prescribing practices rather than establishing absolute clinical superiority of either PARP inhibitor. Collectively, these findings provide an exploratory framework for investigating vulnerability-associated treatment heterogeneity, although these findings are hypothesis-generating and prospective investigation is required before any clinical implications can be considered.

The OVS, an integrated surrogate construct incorporating routine hematologic, hepatic, and renal function indicators, was designed to capture baseline physiological reserve. The equal-weighting structure of the OVS reflects a clinically oriented “bottleneck” framework, whereby substantial impairment in any single physiological domain may independently constrain treatment tolerance. Equal weighting was intentionally adopted to preserve interpretability and reduce the risk of overfitting within a moderate-sized observational cohort. Within the causal inference framework, OVS was modeled exclusively as a prespecified effect modifier to evaluate heterogeneity in the relative treatment effects of olaparib versus niraparib, and was intentionally excluded from the confounder adjustment set to avoid overadjustment and preserve interpretability of the heterogeneous treatment effect estimand (39). To reduce the risk of over-adjustment and multicollinearity, covariate selection was guided by clinical structure and causal relevance rather than univariate statistical screening alone. Accordingly, the present OVS specification should be interpreted as a clinically structured proxy of physiological reserve rather than a formally optimized prognostic scoring system. Individual thresholds were defined according to established clinical guideline recommendations and institutional laboratory reference standards rather than being empirically tuned to the study dataset. Consequently, the current specification may require recalibration and local validation when applied to populations with different demographic characteristics, laboratory reference ranges, or regional clinical practices. Notably, the modest Harrell’s C-index (0.622) and limited KM survival gradient observed for OVS are not inconsistent with its role as an effect modifier. A variable may demonstrate limited independent prognostic discrimination while still meaningfully modifying the relative effectiveness of competing treatments, as these two properties reflect distinct statistical estimands. The observed pattern—where OVS showed limited prognostic value but significant treatment-by-OVS interaction (P = 0.006)—is therefore internally coherent and consistent with the prespecified design in which OVS was modeled exclusively as an effect modifier rather than a prognostic prediction tool.

In patients with low organ vulnerability (OVS 0–1, n = 479), olaparib was associated with a modest relative PFS advantage compared with niraparib (HR = 0.744, 95% CI: 0.569–0.972, P = 0.030), accompanied by only limited separation of the Kaplan–Meier curves. In contrast, among patients with high organ vulnerability (OVS 2–3, n = 125), the relative association favoring olaparib appeared substantially stronger (HR = 0.363, 95% CI: 0.226–0.584, P < 0.001), with earlier divergence of the survival curves and a steeper initial decline observed in the niraparib group during the first 40 months of follow-up. Although these findings should not be interpreted mechanistically, they may be consistent with differences in treatment tolerance or treatment persistence among physiologically vulnerable patients. G-computation analyses demonstrated a similar pattern of heterogeneity, supporting the presence of differential comparative effectiveness across vulnerability strata. The exploratory machine learning–based heterogeneity analysis further identified variation in estimated individual treatment response differences across the cohort. While the majority of estimated response differences favored olaparib, a subset of patients demonstrated predicted relative benefit associated with niraparib. This subgroup may represent patients with distinct biological characteristics; however, the present data are insufficient to characterize this subgroup or to identify specific clinical or molecular determinants of differential treatment response (18, 19, 46).

The present OVS framework nonetheless has important limitations. It does not account for potentially differential contributions of individual organ systems or biomarkers, nor does it exhaustively evaluate all possible weighting schemes or threshold combinations. Although multiple sensitivity analyses demonstrated directional consistency across alternative OVS specifications, the construct may require recalibration and external validation when applied to populations with different demographic characteristics, laboratory reference ranges, or regional clinical practices. Future multicenter studies incorporating larger datasets and data-driven optimization strategies may further refine the weighting structure and improve generalizability.

A benchmark analysis based on BRCA mutation status was conducted to evaluate whether the proposed analytical framework could reproduce a well-established prognostic association under real-world data conditions, rather than to imply a manipulable intervention on germline status (1, 2, 4, 5). Using IPTW to improve baseline comparability between BRCA-mutated and BRCA wild-type groups, the estimated associations remained directionally consistent with prior clinical evidence across multiple analytical strategies. In the CCA, the estimated hazard ratio was 0.679 (95% CI: 0.484–0.954; P = 0.026). MI, implemented as a pre-specified component of the analytical framework for consistency with the primary analysis, yielded nearly identical effect estimates (HR = 0.685, 95% CI: 0.490–0.959; P = 0.028), indicating minimal sensitivity to missing data handling in this benchmark setting. Similar patterns were observed in the platinum-sensitive subgroup (HR = 0.670, 95% CI: 0.476–0.943; P = 0.022), supporting the robustness of the observed association across clinically relevant population restrictions. In addition, IPTW substantially improved covariate balance, with all SMDs reduced to below 0.1 after weighting and adequate overlap observed in propensity score distributions, supporting the plausibility of conditional exchangeability with respect to measured covariates and the positivity assumption. Collectively, these findings indicate that the proposed analytical framework yielded estimates consistent with established clinical evidence under structured confounder adjustment, thereby supporting the internal consistency and operational robustness of the analytical pipeline within the present observational setting.

This internal validation underscores the importance of addressing missingness in real-world data. Missing baseline covariates were handled using both complete-case analysis and multiple imputation, reflecting a trade-off between potential selection bias and statistical efficiency (53, 54). The consistency in effect direction across analytical strategies supports the robustness of the observed associations, while differences in statistical significance highlight the sensitivity of inference to data completeness and modeling assumptions. The explicit categorization of unknown BRCA/HRD status as a distinct “Unknown” group acknowledges that biomarker data in real-world settings are frequently missing not at random, often reflecting clinical and socioeconomic constraints such as rapid disease progression, insufficient tissue availability, or the financial burden of high-cost genomic testing. By employing the Missing Indicator Method rather than imputation, we preserved the integrity of the full cohort and captured the inherent characteristics of untested patients (13). Nevertheless, this approach implies that the “Unknown” group may represent a distinct clinical phenotype, which may introduce residual confounding.

This study addresses several methodological limitations of conventional observational analyses. Prior studies have predominantly relied on standard Cox regression or predictive modeling approaches, primarily focusing on factors associated with PFS rather than exploring potential heterogeneity in the relative effectiveness of different treatments (8, 24). In contrast, the present study integrated G-computation-based counterfactual standardization and an exploratory machine learning–based heterogeneity analysis within a structured causal inference framework (14, 17, 45, 55). The validity of these analyses relies on standard causal inference assumptions, including conditional exchangeability with respect to measured covariates, positivity, and consistency. Although residual unmeasured confounding cannot be excluded in real-world observational settings, E-value sensitivity analysis suggested that relatively strong unmeasured confounding would be required to fully explain the observed treatment-by-OVS interaction.

The absence of systematically recorded treatment-related toxicity data represents an important practical limitation of the present study. Nevertheless, baseline laboratory indicators reflecting hematologic, hepatic, and renal function may partially capture underlying physiological susceptibility to treatment intolerance. For example, lower baseline hemoglobin or platelet levels may reflect reduced bone marrow reserve, whereas elevated creatinine or bilirubin levels may indicate impaired metabolic or excretory capacity (39). These physiological characteristics could potentially influence dose intensity, treatment continuity, or cumulative drug exposure, thereby indirectly affecting clinical outcomes. In this context, patients with higher baseline OVS may have been more vulnerable to treatment-related toxicity, which could partially contribute to the observed differences in relative treatment patterns between PARP inhibitors. The higher incidence of niraparib-associated Grade 3/4 thrombocytopenia reported in the NOVA trial is directionally compatible with this interpretation (5, 6, 25, 38). However, these observations should be interpreted as exploratory and hypothesis-generating rather than definitive mechanistic conclusions.

Another practical challenge in this study was the incomplete availability of BRCA/HRD testing, reflecting common real-world clinical constraints. Molecular testing appeared more frequently performed among physiologically vulnerable patients, potentially reflecting clinical tendencies toward more comprehensive evaluation in patients with complex treatment considerations. Despite potential indication bias, the association between OVS and relative treatment patterns remained directionally consistent across molecular subgroups, suggesting that the observed heterogeneity was not entirely explained by biomarker status alone. The observed patterns within the “Unknown” subgroup may additionally reflect both clinical selection processes and underlying patient heterogeneity (26, 37, 56, 57).

Several limitations should be acknowledged. First, although the primary analysis was supplemented by an independent external validation cohort, the study remained retrospective and observational in nature, and both cohorts were derived from regionally related clinical settings. Therefore, the generalizability of the findings to other healthcare systems, ethnic populations, and treatment environments remains uncertain (8, 24). Second, despite multiple internal robustness analyses and external qualitative replication, the current OVS framework should still be interpreted as an exploratory and clinically structured physiological construct rather than a fully optimized predictive scoring system. The construct incorporated partially heuristic design choices, including equal weighting of organ-system components and predefined threshold selection based on locally adopted clinical guideline standards. Although alternative specifications demonstrated directionally consistent findings, the present study did not exhaustively evaluate all possible weighting schemes, biomarker combinations, or threshold definitions. Future multicenter studies with external calibration and data-driven optimization strategies may further refine the construct and improve generalizability. Third, the absence of longitudinal toxicity data and patient-reported outcomes precluded direct assessment of treatment tolerability, dose modification patterns, and quality-of-life differences between PARP inhibitors (28). Finally, as with all observational causal inference analyses, the validity of the estimated effects depends on assumptions including conditional exchangeability with respect to measured covariates, positivity, and consistency, and residual unmeasured confounding cannot be completely excluded despite sensitivity analyses (16, 40, 41).

In summary, by benchmarking the analytical framework against established molecular associations, this study suggests that baseline physiological vulnerability may be associated with heterogeneity in the relative effectiveness of PARP inhibitors under real-world treatment conditions. While the observed treatment-by-OVS interaction was consistent across multiple sensitivity analyses and directionally reproduced in an independent cohort, the OVS should be regarded as an exploratory and hypothesis-generating construct; further prospective validation and independent external replication are required before any clinical application can be considered. These findings should be interpreted as exploratory and hypothesis-generating rather than practice-changing evidence. More broadly, the integration of molecular tumor characteristics with host physiological reserve may provide a complementary multidimensional framework for investigating treatment heterogeneity in real-world oncology populations.

## Data Availability

The datasets generated and analyzed during the current study are not publicly available because they contain potentially identifiable patient information. De-identified data may be made available by the corresponding author upon reasonable request and subject to institutional approval. The complete analytical workflow, including preprocessing, causal inference modeling, sensitivity analyses, and figure generation scripts, is publicly available at: https://github.com/AllrightChou/ovarian-parpi-ovs-analysis.
