# Supplementary material for: Progression-free survival outcomes of PARP inhibitors in ovarian cancer: an exploratory analysis of treatment heterogeneity based on organ vulnerability
Source: Front Oncol. 2026 Jul 3;16:1768842. doi: 10.3389/fonc.2026.1768842 (PMC13375718; doi:10.3389/fonc.2026.1768842)
Supplement: Supplementary file 1 [file Table1.docx]

Supplementary Material

### Supplementary Results S1. Covariate Balance Diagnostics for the BRCA Benchmark Analysis

Inverse probability of treatment weighting (IPTW) substantially improved baseline comparability between BRCA-mutated and BRCA wild-type groups in the benchmark analysis. Before weighting, several covariates demonstrated moderate imbalance, with the largest standardized mean differences (SMDs) observed for FIGO stage I (SMD = 0.167) and FIGO stage II (SMD = 0.155), followed by Treatment: Second-line (SMD = 0.153). The largest post-weighting SMD was observed for FIGO Stage IV (SMD = 0.083), which remained below the prespecified threshold of 0.1. This residual imbalance is likely attributable to the small number of Stage IV patients in the benchmark subcohort, which limits the precision of SMD estimation for this category. After IPTW adjustment, all absolute SMDs were reduced to below the prespecified threshold of 0.1, indicating adequate post-weighting covariate balance and supporting the validity of the weighted comparison framework.

**Table S1.** Standardized Mean Differences Before and After IPTW Adjustment in the BRCA Benchmark Cohort

| Variable | SMD_before | SMD_after |
| --- | --- | --- |
| Age | 0.029 | 0.027 |
| Treatment: Later-line | 0.069 | 0.081 |
| FIGO stage III | 0.089 | 0.067 |
| Treatment: First-line | 0.098 | 0.061 |
| FIGO stage IV | 0.121 | 0.083 |
| Treatment: Second-line | 0.153 | 0.080 |
| FIGO stage II | 0.155 | 0.082 |
| FIGO stage I | 0.167 | 0.065 |

### Supplementary Results S2. Covariate Balance Diagnostics for the Primary IPTW-Weighted Analysis

Covariate balance diagnostics demonstrated substantial improvement in baseline comparability between treatment groups following IPTW in the primary analysis cohort. Before weighting, moderate imbalance was observed across several clinical and molecular covariates, particularly FIGO stage, BRCA mutation status, HRD status, and treatment line. Following IPTW adjustment, all absolute SMDs were reduced to below the prespecified threshold of 0.1, indicating adequate post-weighting balance and supporting the appropriateness of the weighted causal inference framework for subsequent heterogeneity analyses.

**Table S2.** Standardized Mean Differences Before and After IPTW Adjustment in the Primary Analysis Cohort

| Variable | SMD_before | SMD_after |
| --- | --- | --- |
| FIGO stage III | 0.004 | 0.052 |
| HRD status: Unknown | 0.039 | 0.016 |
| Treatment: Second-line | 0.047 | 0.015 |
| Age | 0.048 | 0.006 |
| Treatment: Later-line | 0.054 | 0.009 |
| Treatment: First-line | 0.078 | 0.008 |
| FIGO stage IV | 0.090 | 0.044 |
| BRCA mutation: Unknown | 0.091 | 0.001 |
| FIGO stage II | 0.095 | 0.063 |
| BRCA mutation: Mutated | 0.137 | 0.026 |
| HRD status: Negative | 0.149 | 0.027 |
| HRD status: Positive | 0.171 | 0.041 |
| FIGO stage I | 0.191 | 0.053 |
| BRCA mutation: Wild-type | 0.231 | 0.025 |

### Supplementary Results S3. External Validation of OVS-Associated Treatment Heterogeneity

**Table S3.** Baseline Characteristics of the External Validation Cohort. Continuous variables are presented as mean ± standard deviation (SD), and categorical variables as number (percentage).

| Characteristics (Category) | Statistics |
| --- | --- |
| Age (years) | 57.17 ± 9.061 |
| FIGO stage |  |
| Stage I | 1 (1.72%) |
| Stage II | 6 (10.34%) |
| Stage III | 39 (67.24%) |
| Stage IV | 12 (20.69%) |
| BRCA mutation status |  |
| Wild-type | 8 (44.44%) |
| Mutated | 10 (55.56%) |
| Missing | 40 |
| Maintenance drug |  |
| Olaparib | 31 (53.45%) |
| Niraparib | 27 (46.55%) |
| Platinum sensitivity status |  |
| Sensitive | 57 (98.28%) |
| Resistant | 1 (1.72%) |
| Absolute neutrophil count (×10⁹/L) | 4.763 ± 4.029 |
| Absolute lymphocyte count (×10⁹/L) | 1.315 ± 0.46 |
| Hemoglobin (g/L) | 117 ± 15.37 |
| Platelet count (×10⁹/L) | 247 ± 112.01 |
| AST (U/L) | 27 ± 13.23 |
| Urea (mmol/L) | 5.23 ± 1.66 |
| Uric acid (μmol/L) | 297.6 ± 85.81 |
| Creatinine (μmol/L) | 65.95 ± 14.92 |
| Total bilirubin (μmol/L) | 9.792 ± 3.928 |
| PFS (months) | 28.91 ± 20.11 |
| OVS |  |
| 0 | 18 (31.03%) |
| 1 | 30 (51.73%) |
| 2 | 9 (15.52%) |
| 3 | 1 (1.72%) |

Baseline characteristics of the external validation cohort (n = 58) are summarized in Supplementary Table S3. Overall, the clinical composition of the external cohort was broadly comparable to that of the primary cohort, with a predominance of advanced-stage disease and platinum-sensitive patients. The distribution of OVS categories was also generally similar to that observed in the derivation dataset, although the external cohort was substantially smaller.

Using the same predefined OVS framework and covariate-adjusted analytical strategy as in the primary analysis, the external validation cohort demonstrated a directionally consistent pattern of OVS-associated treatment heterogeneity. As shown in Supplementary Table S4 and Figure S1, the estimated association between olaparib exposure and progression-free survival was comparatively modest among patients with lower physiological vulnerability (OVS 0–1; n = 48; HR = 0.857, 95% CI: 0.628–1.170), whereas a stronger estimated association favoring olaparib was observed among patients with higher physiological vulnerability (OVS 2–3; n = 10; HR = 0.466, 95% CI: 0.243–0.892).

The treatment-by-OVS interaction term also demonstrated a directionally concordant effect estimate relative to the primary analysis, although statistical significance was not reached in the external cohort (interaction HR = 0.544, 95% CI: 0.259–1.139; P = 0.106) (Supplementary Table S4).

These results are interpreted as preliminary assessments of directional reproducibility rather than formal independent replication, and should be considered in the context of the limited sample size and reduced statistical precision of the external cohort.

**Table S4.** IPTW-Weighted Cox Proportional Hazards Model Evaluating Treatment-by-OVS Interaction in the External Validation Cohort

| Variable | HR (95% CI) | P value |
| --- | --- | --- |
| Age (per year) | 1.108 (0.870–1.412) | 0.407 |
| Stage | 1.298 (0.962 – 1.752) | 0.088 |
| Treatment (Olaparib vs Niraparib) | 0.856 (0.639−1.149) | 0.298 |
| OVS | 1.145 (0.846–1.550) | 0.381 |
| Treatment × OVS interaction | 0.544 (0.259–1.139) | 0.106 |

Note: The treatment-by-OVS interaction term in the external validation cohort demonstrated a directionally concordant but statistically non-significant effect estimate (interaction HR = 0.544, P = 0.106), consistent with the reduced statistical power of the smaller external sample.


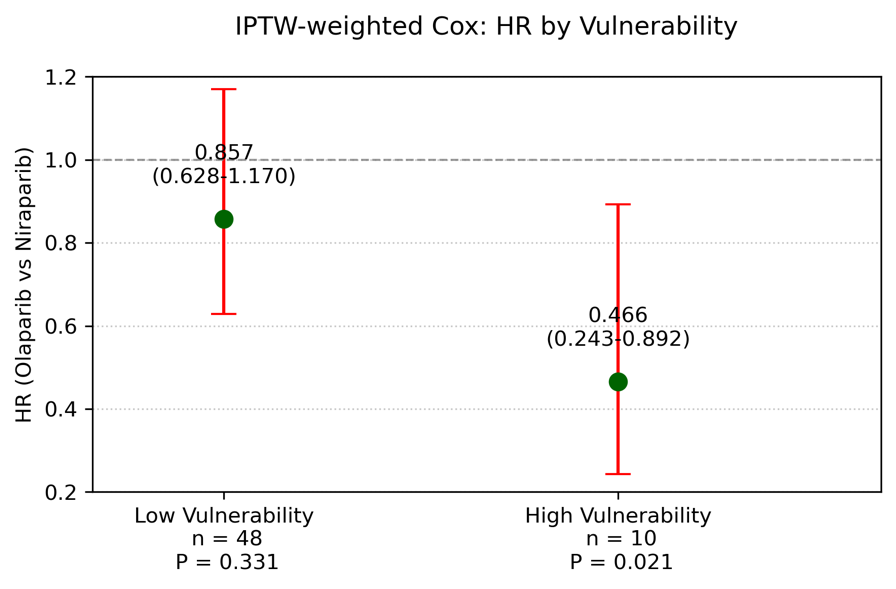


**Figure S1.**  Hazard Ratio Estimates Comparing Olaparib Versus Niraparib Across OVS Strata in the External Validation Cohort. The high-vulnerability stratum in the external cohort comprised only 10 patients, and the corresponding stratum-specific hazard ratio estimate should therefore be interpreted with particular caution given the limited sample size and resulting model instability.

### Supplementary Results S4. Internal Consistency and Robustness Assessment of the OVS Construct

To evaluate the internal coherence of the OVS construct as a representation of increasing physiological vulnerability, OVS-stratified Kaplan–Meier survival curves were examined across all four OVS categories (0, 1, 2, and 3) in the full cohort (n = 604). As shown in Figure S2, a directional gradient in PFS outcomes was observed across increasing OVS strata (log-rank P = 0.011), with patients in the OVS 0 stratum exhibiting the longest estimated PFS. While the magnitude of survival differences across intermediate strata was modest and the OVS 3 curve should be interpreted with caution given the small stratum size (n = 20), the overall directional pattern is consistent with the construct's intended representation of increasing baseline physiological vulnerability.

Importantly, the modest degree of prognostic discrimination observed for OVS is not inconsistent with its role as a prespecified effect modifier. A variable may exhibit limited independent prognostic value while meaningfully modifying the relative effectiveness of competing treatments, as these two properties reflect distinct statistical estimands. The Harrell's C-index for OVS (0.622) and the modest KM survival gradients are therefore interpreted as supportive of construct coherence rather than evidence against the OVS framework, and are consistent with the study's prespecified design in which OVS was modeled exclusively as an effect modifier rather than a prognostic prediction tool.


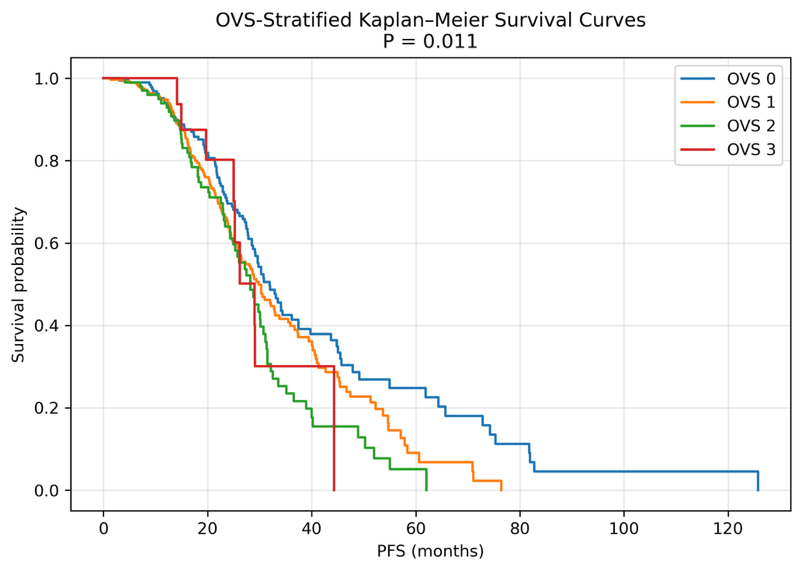


**Figure S2.** OVS-stratified Kaplan–Meier survival curves across all four OVS categories in the full cohort (n = 604). Log-rank P = 0.011. OVS 3 stratum (n = 20) should be interpreted with caution due to limited sample size.

Beyond construct coherence, the robustness of the OVS framework was further evaluated using alternative organ-level vulnerability definitions and OVS dichotomization thresholds, as detailed below. Results are summarized in Supplementary Table S5.

In the primary OVS construct, an organ system was classified as vulnerable if at least one predefined indicator exceeded the corresponding abnormality threshold. Sensitivity analyses subsequently evaluated two progressively stricter organ-level definitions: (I) requiring at least two abnormal indicators within an organ system to classify that organ as vulnerable; and (II) requiring all indicators within an organ system to exceed predefined thresholds.

Within the "≥ 2 abnormal indicators" framework, additional analyses were performed using alternative definitions of high physiological vulnerability, including OVS ≥ 1, OVS ≥ 2, and OVS ≥ 3. Across these specifications, the overall direction of treatment heterogeneity remained consistent with the primary analysis, with olaparib-associated benefit generally becoming more pronounced as vulnerability definitions became increasingly stringent. The strongest interaction effect estimates were observed under the OVS ≥2 threshold within this stricter organ-level framework.

For the most restrictive construct requiring all indicators within an organ system to be abnormal, the observed OVS distribution became substantially compressed, with only OVS scores of 0 or 1 observed in the dataset and relatively few patients classified as OVS = 1. Consequently, analyses under this framework were limited to dichotomization at OVS ≥ 1. Despite the reduced sample size, the estimated treatment-by-OVS interaction remained directionally concordant with the primary analysis.

Under the "≥ 2 abnormal indicators" framework combined with dichotomization at OVS ≥ 3, only two patients met criteria for high physiological vulnerability. Because of extreme subgroup sparsity and resulting model instability, Cox interaction modeling was not considered statistically reliable under this specification, and corresponding estimates were therefore not reported.

**Table S5.** Sensitivity Analyses Using Alternative OVS Construction and Dichotomization Strategies

| OVS specification | High vulnerability definition(n) | Interaction HR (P) | HR in High OVS  (95% CI) |
| --- | --- | --- | --- |
| Primary definition | **OVS ≥2(125)** | **0.488(0.006)** | **0.363(0.226-0.584)** |
| ≥2 abnormal indicators per organ | OVS ≥1(127) | 0.691(0.211) | 0.477(0.290-0.787) |
| ≥2 abnormal indicators per organ | OVS ≥2(7) | 0.204(< 0.001) | 0.135(0.019 – 0.957) |
| ≥2 abnormal indicators per organ | OVS ≥3(2) | - | - |
| All indicators abnormal per organ | OVS ≥1(25) | 0.334(0.016) | 0.225(0.070 – 0.724) |

Overall, these analyses demonstrated that the observed pattern of OVS-associated treatment heterogeneity remained qualitatively stable across multiple alternative OVS construction strategies, and that the construct demonstrated directional coherence as a marker of increasing physiological vulnerability, supporting the internal consistency and robustness of the proposed OVS framework.

### Supplementary Results S5. Validation Analyses Using Individual Physiological Biomarkers

To evaluate whether the observed treatment heterogeneity could be explained by isolated physiological indicators rather than the composite OVS construct itself, additional interaction analyses were performed by sequentially replacing OVS with individual candidate biomarkers incorporated within the broader physiological vulnerability framework. These included all individual indicators comprising the OVS construct (hemoglobin, platelet count, absolute neutrophil count, albumin, total bilirubin, AST, creatinine, and uric acid), as well as additional biomarkers previously associated with PFS (fibrinogen, total bile acids, and thrombin time) and key clinical variables (age and FIGO stage).

As summarized in Supplementary Table S6, none of the individual biomarker-by-treatment interaction models demonstrated statistically significant interaction effects. In contrast, the composite OVS construct retained a significant treatment interaction signal within the primary IPTW-weighted Cox interaction framework. Because biomarker interaction analyses were exploratory and hypothesis-generating, formal multiplicity adjustment was not applied

These findings suggest that the observed treatment heterogeneity is unlikely to be driven by any single physiological parameter alone and instead support the potential value of OVS as an integrated multidimensional representation of baseline physiological vulnerability.

**Supplementary Table S6.** Interaction Analyses Using Individual Baseline Biomarkers in Place of the Composite OVS Construct

| Biomarker | Interaction HR | P value |
| --- | --- | --- |
| Hemoglobin | 0.638 | 0.877 |
| Platelet count | 0.773 | 0.730 |
| Absolute neutrophil count | 0.977 | 0.973 |
| Albumin | 0.998 | 0.995 |
| Total bilirubin | 0.743 | 0.600 |
| AST | 0.837 | 0.556 |
| Creatinine | 0.486 | 0.571 |
| Uric acid | 0.734 | 0.297 |
| Fibrinogen | 0.951 | 0.561 |
| Total bile acids | 0.970 | 0.956 |
| Thrombin time | 0.973 | 0.991 |
| Age | 0.753 | 0.236 |
| Stage | 0.770 | 0.424 |
| OVS | **0.488** | **0.006** |
